# Prof. Austin Flint

**Published:** 1855-07

**Authors:** 


PROF. AUSTIN FLINT, M. D.

We have been favored with an engraved likeness of the above-named gentleman which was gotten up by his professional friends in Buffalo in testimony of their respect for him as a man, for his talents as an editor, and his acquirements as a physician. He has an appointment to one of the chairs in the Institution at Louisville, which he has accepted, and to which place he will soon remove.—Dr. Hunt occupied the position in relation to the Medical Journal once so eminently filled by Dr. Flint, and although too much cannot be said in behalf of Dr. Flint as an editor, still, we are assured the Journal will not lose a tithe of the interest for which it has been heretofore so fully characterized.

---

We have received numerous works which we would be glad to notice, but the want of room forbids. In our next we will give a list of the most important which have come into our hands.

Dr. Bennet at Ft. Desmoine is actively engaged in practice.—Recently he has had a difficult case in the delivery of a double-headed monster, in which one head was placed above another.—The delivery was successfully affected, and the mother did well.

